# Transcriptomic evidence of erythropoietic adaptation from the International Space Station and from an Earth-based space analog

**DOI:** 10.1038/s41526-024-00400-9

**Published:** 2024-05-13

**Authors:** Guy Trudel, Daniel Stratis, Lynda Rocheleau, Martin Pelchat, Odette Laneuville

**Affiliations:** 1 Bone and Joint Research Laboratory, Ottawa Hospital Research Institute, 501 Smyth Road, Ottawa, ON K1H 8L6 Canada; 2 Department of Medicine, Division of Physiatry, Faculty of Medicine, University of Ottawa, 505 Smyth Road, Ottawa, ON K1H 8M2 Canada; 3 Department of Cellular and Molecular Medicine, Faculty of Medicine, University of Ottawa, 451 Smyth Road, Ottawa, ON K1H 8M5 Canada; 4 Department of Biology, Faculty of Science, University of Ottawa, 30 Marie Curie Private Drive, Ottawa, ON K1N 6N5 Canada; 5 Department of Biochemistry, Microbiology and Immunology, Faculty of Medicine, University of Ottawa, 451 Smyth Road, Ottawa, ON K1H 8M5 Canada

**Keywords:** Molecular medicine, Molecular biology

## Abstract

Space anemia affects astronauts and the underlying molecular alterations remain unknown. We evaluated the response of erythropoiesis-modulating genes to spaceflight through the analysis of leukocyte transcriptomes from astronauts during long-duration spaceflight and from an Earth model of microgravity. Differential expression analysis identified 50 genes encoding ribosomal proteins with reduced expression at the transition to bed rest and increased during the bed rest phase; a similar trend was observed in astronauts. Additional genes associated with anemia (15 genes), erythrocyte maturation (3 genes), and hemoglobin (6 genes) were down-regulated during bed rest and increased during reambulation. Transcript levels of the erythropoiesis transcription factor GATA1 and nine of most enriched erythrocyte proteins increased at reambulation after bed rest and at return to Earth from space. Dynamic changes of the leukocyte transcriptome composition while in microgravity and during reambulation supported an erythropoietic modulation accompanying the hemolysis of space anemia and of immobility-induced anemia.

## Introduction

Space anemia was identified since the very first men returned from space and still affects astronauts today. Reduced red blood cell (RBC) and hemoglobin concentrations, and hematocrit are emblematic observations made in astronauts in the weeks after spaceflight^1,2^. The paucity of astronauts samples and the constrains for assessing hematological parameters from fresh blood in space limit our understanding of RBC modulation in microgravity. Prolonged bed rest, with a −6° head down tilt (HDT), serves as an Earth model of microgravity and also consistently leads to a mild anemia at the reambulation phase^3,4^. Recently, increased hemolysis measured in astronauts during spaceflight has been identified as a major contributor to space anemia^5^. Enhanced hemolysis was measured through increased markers of hemoglobin degradation: alveolar carbon monoxide, serum iron, and bilirubin^3,5^. In addition, increased reticulocytes in bed rest studies were accompanied by increased EPO levels at reambulation^3,6^. Similarly in space, erythropoietic stimulation was suspected by higher EPO levels at 2 and 6 months in space and increased reticulocytes at landing^5^. The potential augmentation of the erythropoietic activity while in space remains to be investigated.

Methods for the direct assessment of RBC production are invasive requiring bone marrow biopsies to identify and isolate erythroid lineage cells at distinct stages of differentiation^7,8^. Blaber et al. reported accumulation of erythrocytes in the bone marrow of female mice returning from 15 days in space^9^. In the case of enucleated circulating RBC, erythropoiesis-related genes determining the response to HDT bed rest or to space can only be found in RBC precursors in the bone marrow and therefore inaccessible non-invasively. Alternative approaches such as the spaceflight-specific transcriptome could provide valuable information about the activity of cellular processes in general and erythropoiesis in particular^10^. Studies on pulmonary hypertension, juvenile arthritis, malaria, COVID-19 infection and mountaineering used peripheral leukocytes to probe for gene expression related to erythroid changes associated with anemia or hypoxia^11–15^. With regards to space exposure, the longitudinal NASA Twins Study reported global gene expression changes during long-duration spaceflight and impact on multiple systems^16^. Transcriptome changes in astronauts’ T lymphocytes were confirmed in a global analysis of transcript composition derived from hundreds of blood samples collected during and after spaceflight and retrieved from the NASA’s GeneLab platform^10,17^.

In the current study, we investigated the bone marrow erythropoietic response to microgravity from the leukocyte transcriptome of participants to a HDT bed rest study and of astronauts on ISS missions. The transcriptome composition measured before, during and after bed rest/space exposure was analyzed at specific moments: the transition from ambulatory to the HDT bed rest, the status after 30 and 60 days of HDT bed rest, the transition from prolonged HDT bed rest to reambulation and the recovery from HDT bed rest after 30 days in reambulation. Time-specific differences for the astronauts included the transition from Earth to space, the status after ~2 to 6 months in space, the transition from space to reambulation on Earth, and the recovery from space missions up to one year after reambulation. Our hypothesis was that increased erythopoietic-related gene expression would accompany moments of increased hemolysis and RBC production: during bed rest/in space, and at reambulation after bed rest/return to Earth; the former because of increased hemolysis and the latter to overcome anemia of immobility/space anemia.

## Results

### Transcriptome response to HDT bed rest - BDC vs HDT2

**Fig. 1:**
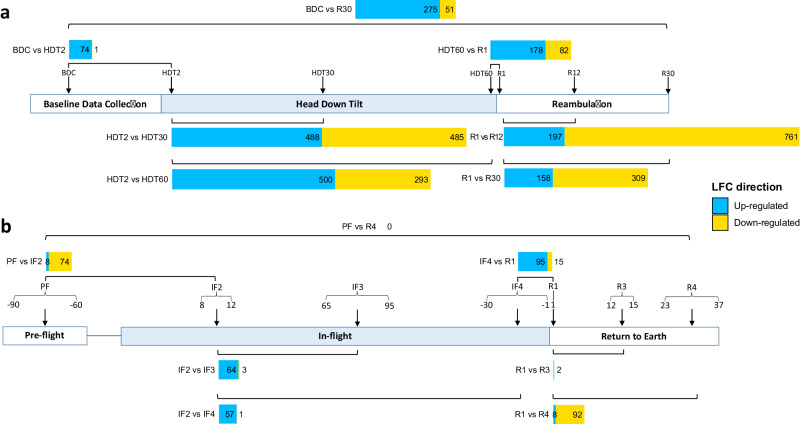
Differentially expressed protein-coding genes at selected time points from the bed rest HDT and astronaut studies. **a** The gene profiles are from Supplementary Material Table 2a, where bed rest and reambulation timepoint comparisons were combined into gene profiles: HDT2 vs HDT30/HDT60 and R1 vs R12/R30. The 3 study phases indicated in the study timeline: BDC (baseline data collection); HDT (head down tilt −6°); R (reambulation). Arrows indicate blood collection days used for transcriptome comparisons. Square brackets indicate timepoints comparisons: BDC, HDT (Day), and R (Day) for the identification of differentially expressed genes. **b** The gene profiles are from Table 2b and included timepoints comparisons: PF vs IF2, IF2 vs IF3, IF2 vs IF4, IF4 vs R1, R1 vs R3, and R1 and R4. The 3 phases of the astronaut study indicated in the study timeline: PF (pre-flight); IF (in-flight); R (return to Earth). Arrows and curly brackets indicate, respectively, blood samples and collection windows of time used in the analysis: PF (range in days), IF (range in days), and R (range in days). Square brackets indicate transcriptome comparisons to identify differentially expressed genes between 2 time points. Horizontal bars indicate the number of up-regulated protein-coding genes (blue) and down-regulated genes (yellow).

The number of differentially expressed protein-coding genes identified from each selected timepoint comparison of bed rest are reported in Supplementary Material Table 2a. On Day 2 of the transition to HDT bed rest, 75 protein-coding genes were differentially expressed compared to baseline (BDC vs HDT2). Up-regulated genes dominated the transition to bed compared to down-regulated genes; 74 genes vs 1 respectively (Fig. 1a). GO term enrichment analysis of these 75 genes resulted in 18 terms mainly describing synaptic transmission and the nervous system (7 terms) as well as immunity and inflammation (5 terms) (Supplementary Material Fig. 1 and Supplementary Information). From the 18 GO terms, 29 protein-coding genes mapped to at least one term and all were up-regulated. The top 10 GO terms with the highest enrichment factors represented in the list of differentially expressed genes between BDC and HDT2 displayed in Supplementary Material Fig. 1a and none related to erythrocytes.

After 30 days in bed, expression changes were far greater (973 genes between HDT2 vs HDT30; and 793 genes between HDT2 vs HDT60) compared to the differences between Days 1 and 2 of bed rest (6 genes between HDT1 vs HDT2) (Supplementary Material Table 2a). Comparing the HDT2 vs HDT30/60 gene lists resulted in 1,228 protein-coding genes, of which 667 were up-regulated and 561 were down-regulated (Supplementary Material Fig. 1a). The biological processes represented by the HDT gene profile were yielded 4 statistically significant GO terms: “ribosomal subunit assembly” (8 genes), “cytoplasmic translation” (58 genes), “proton motive force-driven mitochondrial ATP synthesis” (17 genes), and “nitric oxide biosynthetic process” (15 genes) with no RBC-related GO term (Supplementary Material Fig. 1a). From these four GO terms, 90 genes mapped to at least one term, of which 77 genes were up-regulated (Supplementary Information).

### Transcriptome response to reambulation from HDT bed rest - HDT60 vs R1

The transition from HDT bed rest to reambulation was characterized by the differential expression of 260 protein-coding genes at Day 1 and 50 genes at Day 2 compared to HDT60 (Supplementary Material Table 2a). The number of up-regulated genes was higher than down-regulated genes; 178 genes for HDT60 vs R1 and 38 genes for HDT60 vs R2, compared to 82 and 12 down-regulated protein-coding genes, respectively (Supplementary Material Table 2a). Among the 68 enriched GO terms: “blood coagulation” and “fibrin clot formation” were represented by increased expression of genes THBD, F12, FN1 and decreased expression of GP1BA4 (Supplementary Material Fig. 1b). Additional enriched GO terms included cell-mediated immune processes and inflammation (7 terms) and the cardiovascular system (4 terms) (Supplementary Material Fig. 1b). From these GO terms, 31 genes mapped to at least one GO term, of which 21 were up-regulated. Terms related to erythrocytes were not identified in the gene enrichment analysis.

During the reambulation phase, most changes occurred between R1 and R12 and identified 958 genes including 197 up-regulated genes and 761 down-regulated protein-coding genes. Comparing Day 1 and Day 30 of reambulation identified 467 genes including 158 up-regulated genes and 309 down-regulated. Combining the two reambulation gene lists resulted in 1,110 genes differentially expressed between R1 vs R12/R30 (Supplementary Material Fig. 1a). GO term enrichment generated 88 terms describing humoral immunity and inflammation (7 terms) among the top 10 most enriched GO terms (Supplementary Material Fig. 1b). From these GO terms, 74 genes mapped to at least one GO term, of which 61 were down-regulated. Erythrocyte-related terms did not feature among the most enriched GO terms represented in the list of differentially expressed genes comparing R1 and R12/R30 transcriptomes.

### Transcriptome recovery from baseline

To evaluate transcriptome recovery from 60 days of HDT bed rest, we compared transcriptomes after 30 days of reambulation to baseline. The transcriptome composition did not return to baseline and 326 protein-coding genes remained differentially expressed including 275 up-regulated and 51 down-regulated genes (BDC vs R30) (Supplementary Material Table 2a and Fig. 1a). GO term enrichment analysis resulted in 17 terms including 6 related to red blood cells of which were: “oxygen transport” (HBB, HBG2, HBQ1, HBM, HBD, HBZ), “hemoglobin metabolic process” (SLC25A37, ALAS2, EPB42, AHSP, SLC6A9) and erythrocyte development (ARID4A, DMTN, FAM210B, TRIM58, ALAS2, SLC4A1, EPB42) (Supplementary Material Fig. 1b). From the enriched GO terms, 56 genes mapped to at least one term, of which 48 were up-regulated.

### Erythropoiesis-related genes

**Fig. 2:**
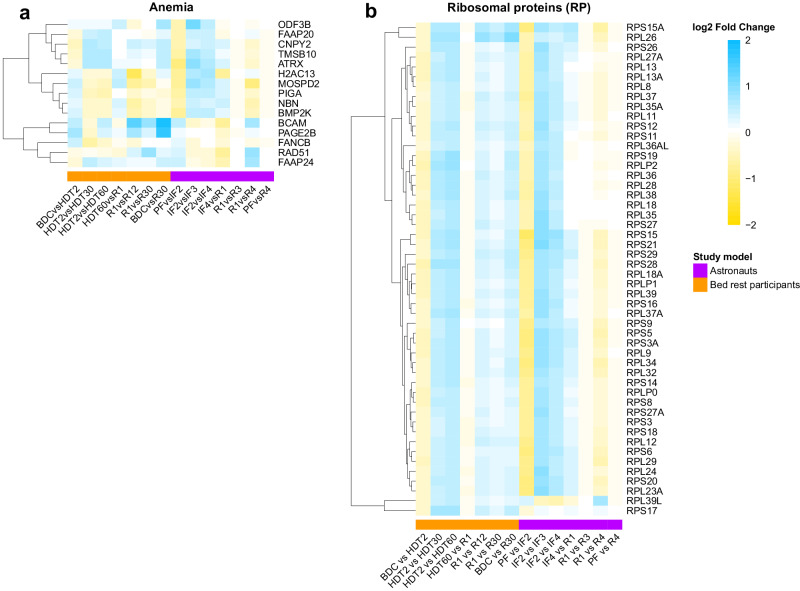
Anemia-related genes and ribosomal proteins coding genes represented among the differentially expressed genes. Heatmaps display the log2 fold change (LFC) values for genes related to anemia (**a**) and of the ribosomal proteins (RP) family; RPL and RPS (**b**). Cell colors represent the LFC values for any given timepoint comparison, where blue indicates up-regulation and yellow indicates down-regulation (color scale). Bed rest data represented in the columns above the orange bar and astronaut data above the purple bar. HUGO Nomenclature Committee (HGNC) symbols were used to name genes. Dendrograms grouped genes with similar temporal profiles of changes. This Figure shows coordinated, en bloc change of RP gene expression at phase transitions to and from bed rest and to and from space.

**Fig. 3:**
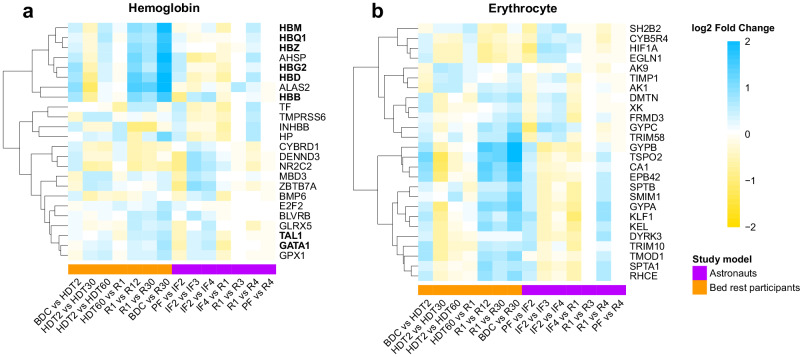
Differential expression of hemoglobin and erythrocyte-related genes during bed rest HDT and spaceflight. Heatmaps display the log2 fold change (LFC) values for genes associated with hemoglobin (**a**) and erythrocytes (**b**). Cell colors represent the LFC values for any given timepoint comparison, where blue indicates up-regulation and yellow indicates down-regulation (color scale). Bed rest data represented in the columns above the orange bar and astronaut data above the purple bar. HUGO Nomenclature Committee (HGNC) symbols were used to name genes. Dendrograms grouped genes with similar temporal profiles of changes. Hemoglobin genes (HB) and transcription factors regulating erythropoiesis GATA1 and Tal1 indicated in bold. This figure shows coordinated change in hemoglobin and erythrocyte-associated gene expression at phase transitions to and from bed rest and to and from space.

To search genes related to erythropoiesis, we interrogated the lists of all differentially expressed genes from the bed rest and the space studies highlighted in Supplementary Material Table 2. The profiles of expression for anemia, erythrocyte proteins, and hemoglobin-related genes at specified times for both bed rest and astronaut studies are displayed as heatmaps (Figs. 2, 3). The following statistically significant differentially expressed genes during bed rest (HDT2 vs HDT30/60) have also been associated with different types of anemia including: Diamond-Blackfan anemia (TMSB10), alpha thalassemia (ATRX), Fanconi anemia (MOSPD2, FAAP24, FANCB, CNPY2, H2AC13), microcytic anemia (TMPRSS6). Genes associated with anemic conditions were differentially expressed during the bed rest study (Fig. 2a). Genes BCAM linked to congenital dyserythropoietic anemia type IV and PAGE2B associated with recessive X-linked sideroblastic anemia were both up-regulated at R30 compared to baseline. None of these associations reached statistical significance in the astronaut cohort (Fig. 2a).

Additional anemia-related genes identified included a large number of ribosomal protein genes; RPS and RPL. Mutations in one of the many RPS and RPL genes cause Diamond-Blackfan anemia. A heatmap showing the LFCs expression for the 50 ribosomal protein (RP) genes for both studies is presented in Fig. 2b. Remarkably, the expression profiles of all 50 RP genes throughout the bed rest study were the same; they were reduced at the transition to bed (BDC vs HDT2), increased during bed rest (HDT2 vs HTD 30 and HDT2 vs HDT60), unchanged at the transition from bed rest (HDT60 vs R1), at reambulation (R1 vs R12 and R30), and at recovery (BDC vs R30). In astronauts, changes in RP gene expression displayed the same trend as bed rest participants except for RPL39L but did not reach statistical significance (Fig. 2b).

SLC4A1 named erythrocyte membrane protein band 3, displayed the highest LFC of all solute carrier family (SLC) genes on reambulation Day 30 compared to baseline (BDC vs R30). SLC4A1 was also increased during reambulation when comparing R1 and R12/R30, and similarly up-regulated in astronauts between Day 1 and 12 to 15 days after returning to Earth (Supplementary Material Fig. 2a and Table 2). Additional members of the SLC family of genes were detected with 47 differentially expressed genes during the bed rest phase (Supplementary Material Fig. 2a). Changes of SLC gene expression throughout the bed rest and space studies are displayed in a heatmap (Supplementary Material Fig. 2a) and values with statistical significance in Supplementary Material Table 2. SLC genes expression was variable with some but not all members displaying similar profiles. During the bed rest HDT phase, 35 SLC genes were down-regulated and 12 were up-regulated (HDT2 vs HDT30 and HDT60). During the reambulation phase, 30 SLC genes were down-regulated and 17 up-regulated (R1 vs R12/R30). For most SLC genes, expression during spaceflight either decreased during the first days in space (PF vs IF2) and later increased in space (IF2 vs IF3 and IF4) or displayed an increase then decrease profile (Supplementary Material Fig. 2a).

A third family of genes linked to erythropoiesis differentially expressed during the bed rest encodes for mitochondrial proteins and represented by 44 genes: NADH ubiquinone oxidoreductase (NDUF; 12 genes), cytochrome C oxidase (COX; 6 genes), ATP synthase (ATP5; 6 genes), mitochondrial ribosomal proteins (MRPL/S; 11 genes), and mitochondria genes (MT-; 9 genes) (Supplementary Material Fig. 2b). Mitochondrial transcripts originated from both nuclear and mitochondrial genomes (MT, 9 genes). Expression of most mitochondrial genes in the nuclear genome were increased during bed rest (HDT2 vs HDT30/HDT60) and during reambulation (R1 vs R12 and R30) except for 2 genes, COX6B2 and NDUFV2 (Supplementary Material Fig. 2b). Genes encoded in the mitochondria genome decreased during the bed rest phase (HDT2 vs HDT30 and HDT60) and majority increased during reambulation (R1 vs R12 and R30) (Supplementary Material Fig. 2b). Mitochondrial genes were unchanged or displayed small variations throughout spaceflight, which did not reach statistical significance (Supplementary Material Fig. 2b). The dominating trend of mitochondrial gene expression was a decreased expression at the transition to space, increased expression while in space and unchanged after return to Earth.

The expression of two key transcription factors driving erythrocyte differentiation, GATA1 and TAL1, increased at R30 compared to BDC (Fig. 3a). Genes regulated by GATA1 include 6 hemoglobin genes; HBB, HBD, HBG2 from the beta globin locus and HBM, HBQ1, HBZ from the alpha globin locus. All six Hb genes displayed the same profile; up-regulated at transition to bed (BDC vs HDT2), down-regulated during the bed rest phase (HDT2 vs HDT30), up-regulated during the reambulation phase (R1 vs R12/30), and remained up-regulated at 30 days of reambulation compared to baseline (BDC vs R30) (Fig. 3a). TRIM58 gene participates in the erythroblast enucleation process and was increased at R30 compared to baseline (Fig. 3b). An additional GATA1-targeted gene identified from the leukocytes’ transcriptome was FAM210B, a mitochondrial membrane protein abundantly expressed in the later stages of erythroblast development. FAM210B, was up-regulated in bed rest participants during reambulation (R1 vs R12/30) and at recovery (BDC vs R30). Two genes coding for proteins influencing the structure of erythrocytes, TMOD1 and DMTN, decreased during bed rest and increased during reambulation (Fig. 3b). Additional red blood cells proteins identified included ALAS2, EPB42, and SLC4A1 with similar profile of changes; down regulation during the bed rest phase and up-regulation during reambulation (Figs. 2, 3).

### Astronauts’ transcriptome

**Fig. 4:**
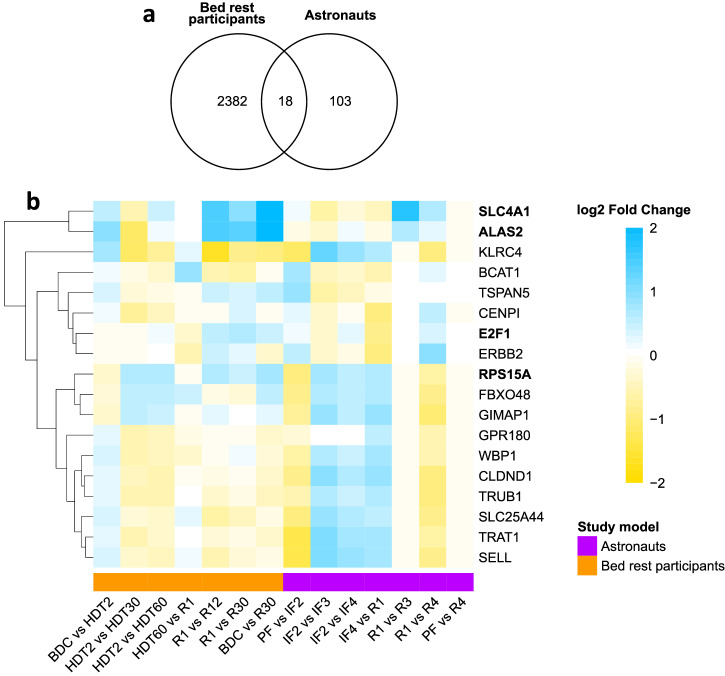
Differentially expressed genes common to participants to bed rest HDT experiments and astronauts. **a** Venn diagram representing the number of differentially expressed genes identified from all timepoint comparisons for bed rest participants, astronauts, and common to both studies. **b** Heatmaps display the log2 fold change (LFC) values for genes common to both studies only. Cell colors represent the LFC values for any given timepoint comparison, where blue indicates up-regulation and yellow indicates down-regulation (color scale). Bed rest data represented in the columns above the orange bar and astronaut data above the purple bar. HUGO Gene Nomenclature Committee (HGNC) symbols were used to name genes. Dendrograms grouped genes with similar temporal profiles of changes. The four erythropoiesis-related genes are bolded.

The number of differentially expressed genes for all comparisons identified during spaceflight was lower compared to all bed rest comparisons; 121 vs 2400 genes (Fig. 4a). After 8 to 12 days in space, the expression of 74 protein coding genes were down regulated and 8 genes were up regulated (PF vs IF2) (Supplementary Material Table 2b). In-flight, genes up-regulation dominated compared to down-regulated protein-coding genes; IF2 (average 10 days in space) vs IF3 (average 80 days in space) identified 64 vs 3 genes and IF2 (average 10 days in space) vs IF4 (average 15 days before returning on Earth): 57 vs 1. The return to Earth after ~6 months in space was characterized by an up-regulation of expression: 95 genes, with 15 down-regulated genes (IF4 or average 13.5 days in space vs R1). Recovery from space was characterized by 92 down-regulated genes and 8 up-regulated genes (R1 vs R4 or average 30 days after return to Earth) and only 2 up-regulated genes (R1 vs R3 or average 13.5 days). The astronauts’ leukocyte transcriptome returned to pre-flight composition 30 ± 7 days (R4) after the return on Earth (Supplementary Material Table 2b).

To compare bed rest and astronaut transcriptomes, we identified differentially expressed genes common to both studies. Among the bed rest 2,400 protein-coding genes and 121 genes identified from the astronaut study, we identified 18 common genes to both studies (Fig. 4a). LFC values of the genes common to both studies at specified timepoints are visualized in a heatmap (Fig. 4b). Four erythropoiesis-related genes were common to both studies; SLC4A1, ALAS2, E2F1, and RPS15A, and displayed similar profiles of changes in both cohorts (Fig. 4b).

## Discussion

The most prominent signal supporting stimulated erythropoietic activity was the increase expression of 50 members of the RP gene family during the bed rest phase and in-flight. Natural mutations in RP genes produce Diamond Blackfan anemia (DBA); a rare genetic disorder characterized by a paucity of erythroid precursors in the bone marrow and low erythrocyte concentration in blood^18^. DBA patients with a single mutation in one RPs gene have an overall reduction of ribosome levels in their hematopoietic cells and reduced erythroid cells production^19^. Khajuria et al. in an elegant study demonstrated that low ribosome levels selectively impaired erythroid lineage commitment and GATA1 required a high translation rate relative to various master regulators of hematopoiesis^20^. Concomitant to increased RP expression was the increased expression of 33 mitochondria genes during bed rest and 17 during in-flight. Mitochondria are required in the first steps of heme biosynthesis which is crucial for RBCs^21^. The coordinated expression of ribosomal and mitochondrial genes is lost in members of a large DBA family with an intronic mutation in the RPL11 gene and reduced expression of all RP genes while mitochondrial gene expression increased^22^. Individuals participating in the current study are healthy and therefore a coordinated increase of RP and mitochondrial genes during the bed rest phase/in space is expected to support erythropoiesis.

The master regulators of erythroid differentiation, GATA1 and Tal1, transcription factors essential for erythropoiesis both displayed increased expression at recovery in bed rest participants and astronauts^23^. GATA1 induces heme accumulation by directly inducing HB genes expression^24^. In the bed rest study, increased GATA1 transcript at R30 vs BDC was timed with the augmentation of 6 different HB genes; 3 located in the alpha globin locus (HBZ, HBM, HBQ1) and 3 in the beta globin locus (HBG2, HBD, HBB), suggesting a potential contribution for GATA1 in the regulation of both globin loci. Consistent with the different HB genes identified in our study, their corresponding Hb proteins were detected by mass spectrometry in a quantitative analysis of the healthy human RBC proteome^25^. Hb proteins alpha (HbA) and beta (HbB) were the dominant proteins along with lower levels of Hb subunits delta (HbD), fetal gamma (HbG) in addition to embryonic Hb theta (HbQ), zeta (HbZ), and mu (HbM) proteins^25^. Of interest is the suggested hypothesis that exposure to space would trigger HBG genes and the mechanism of globin switch with a preference for fetal globin chains with higher oxygen affinity such as embryonic and fetal hemoglobin^26^. The significant increase of expression of HBG2 gene measured at recovery is consistent with previous reports of post-flight increase relative to pre-flight^17^. Reports of in-flight measurements are limited and results variable; the NASA Twin Study reported decreased HBG1 and HBG2 transcripts relative to pre-flight in CD4 isolated cells and increased in CD8 cells in one astronaut^16,17^. Results from 6 astronauts described both decrease and increase fetal Hb transcripts at various time in space^17^. We report the potential activation of the globin switch mechanism limited to bed rest participants at recovery only and not during the bed rest phase. None of our 14 astronauts’ HbG transcripts underwent a statistically significant change at any time during their missions. Differences of sampling schedules and individual variability combined with the low participants’ number may account for differences between studies. Measures of fetal Hb polypeptides from samples collected in microgravity, to our knowledge not yet available, would provide direct evidence for fetal globin contribution to erythrocyte Hb content in space.

The transcription factor GATA1 regulates hundred of genes whose actions mediate important functions including hematopoietic stem cells differentiation^27^. Our analysis identified differential expression of 50 members of the solute carrier (SLC) family regulated by GATA1. Notably the SLC4A1 gene significantly increased during reambulation after bed rest and after returning to Earth. SLC4A1 is an erythrocyte membrane protein acting as an anion exchanger involved in the chloride/bicarbonate exchanger and carbon dioxide transport from tissues to lungs^27,28^. A second member of the SLC family, identified in both bed rest participants and astronauts, is a mitochondrial solute transporter SLC25A44 transporting branched-chain amino acid into mitochondria^29^. A third mitochondrial solute transporter identified, SLC25A37, functions as an essential iron importer for the synthesis of mitochondrial heme and increased during bed rest^30^. Overall, the differential expression of multiple members of the SLC family support the GATA1 factor-regulated SLC ensemble in establishing the small molecule repertoire required by erythropoietic progenitor cells to efficiently generate erythrocytes during bed rest and in space^27^.

The enrichment analysis identified erythropoiesis-related GO terms only from the recovery (R30 vs BDC) list of differentially expressed genes. Differential expression of many genes from the recovery (R30 vs BDC) erythroid-related GO terms was also measured during the bed rest phase and reambulation indicating increased erythropoiesis-related activity throughout the study. Hematopoietic factors and erythrocyte protein genes (ALAS2, TRIM10, and TSPO2) were up-regulated during the reambulation phase of bed rest. An erythroid-specific mitochondria located enzyme encoded by ALAS2 gene, catalyzes the first step in heme biosynthesis, TRIM10 plays an essential role in the differentiation and survival of terminal erythroid cells, and TSPO2 is required to meet cholesterol demands during erythropoietic differentiation^31–33^. TRIM58 involved in erythroblast enucleation and SMIM1 participating in red blood cell formation were unchanged during bed rest but increased at reambulation^34,35^. The gene TIMP1 stimulates the growth and differentiation of human erythroid progenitors in vitro and increased during bed rest^36^. Reduced expression of KLF1 and DYRK3 was detected at HDT30/60 compared to HDT2 while TIMP1 and ZBTB7A genes increased. The differentiation of hematopoietic stem cells towards erythropoiesis is under the control of different transcription factors, of which Erythroid Krüppel-like Factor (EKLF/KLF1) plays a global role in all aspects of red blood cell formation^37^. Genes regulated by KLF1 included: DMTN, EPB42, GYPA, HBB all increased at R30 compared to baseline.

Additional genes encoding for erythrocyte proteins; CA1, TMOD1, EPB42, and KEL, all decreased during bed rest and increased at reambulation and recovery. The current analysis identified differentially expressed genes encoding nine of the twenty most enriched erythrocyte proteins^25^. Measures of erythrocyte protein levels from bed rest participants and astronauts have been limited to RBC and Hb concentrations and hematocrit performed as part of a complete blood count (CBC) on fresh blood at different time points. In bed rest, Hb concentrations increased at HDT20, HDT49 and HDT60, was unchanged at R1 and, decreased at R13 and at R30 compared to baseline levels^3^. Hemoglobin transcripts and proteins changes measured in the same individuals correlated with adaptation to bed rest and with transition to reambulation. In space, CBC cannot be performed. A rare study collected fresh blood from 31 astronauts while in space that was analyzed on Earth approximately 48 h after collection^38^. As for the bed rest study, RBC concentrations were increased after 2 weeks, 3 and 6 months and Hb concentration only after 2 weeks onboard the ISS. A persistent increase of carbon monoxide elimination, an indicator of hemolysis, was measured at days 5, 11, 30, and 57 of bed rest and at days 5, 11, 64, 157 of long-duration spaceflights^5^. RBC and Hb concentrations must consider plasma volume, a variable changing at transition to and from space. Plasma volume decreased ~15% within the first 24 h in space and was rapidly restored upon returning to Earth^39^. The gene expression analysis considered the fluid shifts and compared samples at transitions: day 1 of bed rest compared to baseline as well as day 1 of reambulation compared to HDT60. Comparisons at later time points of the bed rest and reambulation phases informed adaptation responses. Unchanged Hb transcript levels at phase transitions; day 1 of bed rest and of reambulation; suggest no apparent contribution of fluid shifts to erythropoietic control. After bed rest/landing from space, the full extent of the anemia is uncovered by a reversed fluid shift. Living vertically at Earth-level’s gravity requires a larger RBC mass than when bed ridden or in space and triggers an erythropoietic production in the 3 months following reambulation from bed rest or space to overcome anemia of immobility/space anemia reflected in the leukocyte transcriptomic data.

The transcriptome results add to our current understanding of anemia of immobility and space anemia. In both conditions, increased hemolysis is suggested to be a primary effect of the bed rest position/exposure to space^3,5^. Uncompensated by increased RBC production, increased hemolysis could lead to severe anemia. The increased expression of erythropoietic-related genes after 30 and 60 days of HDT bed rest and after 2 and 6 months in space suggest the stimulation erythropoiesis. The compensatory erythropoiesis stimulation in the bone marrow would prevent the anemia of immobility/space anemia from reaching moderate or severe levels.

Our study bears limitations. Gene expression profiling of peripheral leukocytes is a powerful tool to identify surrogate erythroid markers and direct correlation with bone marrow erythroid precursors was not carried out in the study. Only 3 pre-flight samples were included in the GLM since only 3 of the 14 samples collected at pre-flight passed the quality metrics for sequencing. This results in unbalanced data and increased variability for pre-flight samples resulting in decreased statistical power when testing for gene differential expression. For both studies, we analyzed changes of transcript levels, which represent steady state levels, limiting our ability to comment on changes including transcription, RNA degradation, translation, and post-transcriptional modifications. Important changes happening in between blood draws would have been missed. Bed rest samples were collected on exact days but for astronauts each time point corresponded to a window of days introducing variability. Leukocyte and RNA isolation were not possible on the ISS and blood samples were frozen at −80°C for their journey back to Earth, leading to cell lysis and some RNA degradation. The stringent anthropomorphic and demographic recruitment criteria for each study prevent extrapolation to a wide, heterogeneous or ill population. Importantly, male-only participants to the bed rest study and crew mixed representation with only 3 women astronauts prevented sex-specific comparisons.

## Methods

### Participants

The bed rest Cocktail study recruited twenty healthy male subjects and tested the efficacy of a nutritional intervention to palliate the negative effects of HDT bed rest^40^. Ten participants received daily pills of the cocktail (XXS-2A-BR2 nutrient cocktail; Spiral Cie, Dijon, France) throughout the HDT phase. The current investigation was conducted as a substudy within the “Cocktail” HDT trial and reviewed by the ethics committee at our institution (#20160925-01H).

The MARROW (Marrow Adipose Reaction: Red Or White) study recruited fourteen astronauts, 3 women and 11 men, participating in long missions aboard the ISS^41^.

### Ethics approval and consent to participate

Research ethics approval was obtained from the Comité de protection des personnes Sud-ouest et outre-mer (ID-RCB:2016-A00401-50) and was registered at ClinicalTrials.gov (A New Nutritional Countermeasure to Prevent the Deconditioning Induced by 60 Days of Antiorthostatic Bed Rest (LTBRCocktail, NCT03594799). All MARROW astronauts gave informed consent and were monitored by a medical team at NASA. Ethics approval obtained from the NASA Human Research Multilateral Review (#Pro1283), Johnson Space Center Institutional Review Board (JSCIRB), European Space Agency Medical Board, Japanese Aerospace Exploration Agency, and our institution (#2009646-01H). Participants to the Cocktail HDT bed rest and to MARROW study provided written informed consent to take part in those studies.

### Blood collection, leukocyte isolation, and RNA isolation

For both studies, ten blood samples (~4 mL) collected from each individual after an overnight fast at specific times. For the bed rest study, blood sample collected on specific days: BDC (−12, −11), HDT (1, 2, 30, 60), R (1, 2, 12, 30). For the astronaut study, samples collected over an interval of days; one sample at PF 90 to 60 (average 75) before liftoff, four samples IF onboard the ISS (IF1: 4 to 6 (average 5); IF2: 8 to 12 (average 10); IF3: 65 to 95 (average 80); IF4: 30 to 1 day (average 14.5) before returning to Earth), and five samples upon return to Earth (R) (R1: day 1; R2: 3 to 7 (average 5); R3: 12 to 15 (average 13.5); R4: 23 to 37 (average 30); R5: 335 to 395 (average 365). A total of 200 blood samples were collected from 20 bed rest participants and 139 blood samples were collected from 14 crewmembers during their ISS missions^40,41^.

Leukocyte isolation, RNA extraction, library preparation, and sequencing methods were previously detailed^40,41^. The steps of leukocyte isolation and RNA extraction were performed from fresh blood for the bed rest study and from frozen blood for astronauts using the LeukoLOCK™ total RNA isolation system and the manufacturer’s protocol (#AM1923; ThermoFisher Scientific). Essentially, the leukocyte population was isolated from whole blood by capture on a filter. Isolated leukocytes washed with phosphate-buffered saline, stabilized with the RNAlater solution, and stored at -80°C. RNA isolation from captured leukocytes performed using RNA binding beads, treated with turbo DNase to remove DNA and total RNA used for library preparation and sequencing.

### Library preparation, sequencing, and mapping

Of the 200 samples collected for the bed rest study, 3 from the HDT phase had RIN ≤ 8.0 and were not further analyzed: Cocktail group: B1-3, D1-4, and control group: G1-3. Bed rest RNA sequencing libraries were prepared from 1 μg of total RNA using the Illumina Stranded mRNA Prep kit, and instructions from the manufacturer (#AM1923; ThermoFisher Scientific). Library quality was assessed using the Agilent BioAnalyzer 2100 and 197 samples passed the quality metrics for sequencing, including a concentration above 10 nM. MARROW RNA sequencing libraries were depleted of rRNA using the NEBNext® ribosomal RNA (rRNA) Depletion Kit (Human/Mouse/Rat) (#6310 L, Ipswich, MA, USA) or using the NEBNext® Ultra™ II Directional RNA (rRNA) Library Prep Kit for Illumina® (#E7760L, Ipswich, MA, USA). Library quality was assessed using the Agilent BioAnalyzer 2100, and 72 of the 139 samples passed the quality metrics for sequencing with sample inventory detailed in a previous publication^40,41^.

Libraries (125 pM) from each study were multiplexed and sequenced in two separate runs to generate 100-nt paired-end reads to a depth of ~50 million reads per sample using the Illumina NovaSeq 6000 System (Illumina, San Diego, CA, USA). Quality assessment, library preparation, high-throughput sequencing and de-multiplexing steps were performed at the Genome Quebec Innovation Center (Montreal, Canada). Raw reads analysis was performed in our laboratory. Gene-level mapping of reads were aligned to the publicly available human genome (GRCh38.84) using *TopHat2* or HISAT2 with default parameters^42^. Transcript abundance calculations were performed using *HTSeq* with default parameters (version 0.6.1)^43^. Gene read counts were normalized for sample read depth using *DESeq2’s* median of ratios^44^.

For the bed rest study, read count data for the two baseline timepoints (BDC-12 and BDC-11) from the same participant were averaged to combine samples into one baseline timepoint (BDC)^40^. Genes with mean normalized read counts less than 2 across all samples were excluded from further analysis leaving an expressed transcriptome consisting of 21,419 coding and non-coding genes at any of the nine timepoints: one at BDC, four at HDT bed rest, and four at R to perform differential expression analyses^40^. For the MARROW study, rRNA genes and genes with mean normalized read count less than 45 across all samples were excluded leaving an expression dataset corresponding to 15,410 coding and non-coding genes measured at any of the 10 individual study time points^41^. All transcriptome analyses and result visualizations were conducted in the R environment (https://cran.r-project.org/doc/manuals/r-release/R-intro.html) using packages and custom scripts.

### Differential expression analysis

To test for differential expression between two selected time points for each study, we applied fixed-effect generalized linear modeling (GLM) with the *DESeq2* package in R^44^. First, we analyzed the bed rest transcriptomes by applying three fixed-effect GLMs to test for expression changes between selected time points of the bed rest study and specific to three moments: HDT bed rest, reambulation, and the recovery to baseline after 30 days (Supplementary Material Table 1a). The “replicate” term in the models accounted for the number of biological replicates from each bed rest participant; the “Cocktail” term refers to the assignment of participants to either the control or Cocktail group; the “bed rest”, “reambulation”, and “recovery” variables reflected the inclusion of samples from the selected time points (Supplementary Material Table 1a). For the astronaut study, we applied a fixed-effect GLM to test for expression changes between selected time points and specific to three biological questions relating to spaceflight, return to Earth, and the recovery to pre-flight after 30 ± 7 days (Supplementary Material Table 1b). The “replicate” term in the models accounted for the number of biological replicates from each astronaut while controlling for the confounding variables “sex” and “cumulative lifetime in space”; the “time” variable reflects the selected timepoints (Supplementary Material Table 1b). For both studies, significance testing utilized the Wald’s test on Log2-Fold changes (LFCs); unshrunken LFCs for the bed rest study and shrunken LFCs for the astronaut study using the *ashr* method. Adjustment for multiple comparisons employed independent hypothesis weighting (IHW) to increase statistical power while controlling for false discovery rates (FDR) with the Benjamini–Hochberg correction^45,46^. For the bed rest study, genes with both an FDR adjusted *p*-value < 0.05 and LFC > |0.5| were considered differentially expressed, whereas the astronaut study used an FDR adjusted *p*-value < 0.1 and LFC > |0.5|. Identified differentially expressed genes from both studies were sub-grouped into protein-coding genes using annotations from the *biomaRt* package in R^47^. Further analysis of differentially expressed genes applied to protein-coding genes only.

### Enrichment analysis

Enrichment analyses were limited to the bed rest study and timepoint comparisons consisting of 75 or more differentially protein-coding genes (highlighted in Supplementary Material Table 2). Enrichment analyses were not performed with the astronaut differentially expressed genes due to the low number of genes identified likely caused by the necessary freezing step of blood. Biological functionality was determined with an over-representation analysis (ORA) of gene ontology (GO) terms for each timepoint comparison. First, the *enrichGO()* function within the *clusterProfiler* package in the R environment mapped gene candidates to their associated GO term grouped under “Biological Processes” at any level^48^. Second, one-sided Fisher Exact Tests were applied to identify over-represented GO terms between the mapped differentially expressed gene candidates and the reference list of 21,419 genes. GO terms with FDR adjusted *p*-values < 0.05 were considered statistically significant. Similar GO terms were grouped together based on Lin’s semantic similarity measure using the REVIGO software^49,50^. Up to ten GO terms for each timepoint comparison were displayed in a dot-plot.

### Representation of erythrocyte-related genes

The lists of all differentially expressed genes from both studies were inspected to identify additional gene functions through queries in the GeneCards (https://www.genecards.org/) and NCBI (https://www.ncbi.nlm.nih.gov/gene) databases. Genes relating to anemia, hemoglobin, erythrocyte, and erythropoiesis were mined and their estimated LFC values for selected timepoint comparisons from both studies were displayed in heatmaps using the *pheatmap* package in R.

### Supplementary information

Supplementary Information

Supplemental Material

## Data Availability

The datasets presented in this article are not readily available because of attributability and confidentiality restrictions. Aggregated data to understand and assess the conclusions of this research are available in the figures and supplementary tables. Individual astronaut aggregated data (read count tables and metadata) and code have been deposited in NASA’s Life Sciences Data Archives (LSDA) under dataset name “MARROW payload”. Investigators can request access to the astronaut data at jsc-lsda@mail.nasa.gov.
