# Supplementary material for: Transcriptomic evidence of erythropoietic adaptation from the International Space Station and from an Earth-based space analog
Source: NPJ Microgravity. 2024 May 13;10:55. doi: 10.1038/s41526-024-00400-9 (PMC11091056; doi:10.1038/s41526-024-00400-9)
Supplement: Supplementary file 2 — Supplemental Material [file 41526_2024_400_MOESM2_ESM.pdf]

Supplementary Table 1

a

**Biological question**	**Model**	**Samples (n)**[1]	**Comparison**[2]
1-effect of bed rest?	read counts ~ replicate + cocktail + bedrest + ε	BDC (20) and HDT (77)	BDC vs HDT1<br>BDC vs HDT2<br>HDT1 vs HDT2<br>HDT2 vs HDT30<br>HDT2 vs HDT60<br>HDT30 vs HDT60
2-effect of reambulation?	read counts ~ replicate + cocktail + reambulation + ε	HDT60 (20) and R (80)	HDT60 vs R1<br>HDT60 vs R2<br>R1 vs R2<br>R1 vs R12<br>R1 vs R30<br>R12 vs R30
3-recovery to baseline?	read counts ~ replicate + cocktail + recovery + ε	BDC (20) and R (80)	BDC vs R30

[1]total number of samples used in the 3rd variable of each GLM
[2]selective time point comparisons for each model

b

**Model**	**Samples (n; sex)**[1]	**Biological question**	**Comparison**
read counts ~ replicate + sex + cumulative time in space + time + ε	PF (3; 3 men + 0 women), IF (27; 11 men + 3 women), R (41; 11 men + 3 women)	1-effect of spaceflight?	PF vs IF2<br>IF2 vs IF3<br>IF2 vs IF4
		2-effect of return to Earth?	IF4 vs R1<br>R1 vs R3<br>R1 vs R4
		3-recovery to preflight?	PF vs R4

[1]total number of samples used in GLM.
[2]selective time point comparisons for each biological question.

Supplementary Table 1 | Summary of generalized linear models (GLMs) to identify differentially expressed genes. We interrogated the leukocyte transcriptome of bed rest participants and astronauts with the same 3 biological questions: 1) What is the effect of prolonged exposure to bedrest or to space?; 2) What is the effect of reambulation after prolonged bed rest HDT (60 days) or landing from space (6 months)?; How complete is the recovery from prolonged bed rest and spaceflight? This Table shows the GLM strategies to address the 3 questions for (a) the 60-day bed rest HDT study and (b) astronaut study.

Supplementary Table 2

a

Biological question	Comparison[1]	Differentially expressed protein-coding genes		
		Total	Up-regulated[2]	Down-regulated[3]
1-effect of bed rest?	BDC vs HDT1	0	0	0
	**BDC vs HDT2**	**75**	**74**	**1**
	HDT1 vs HDT2	6	6	0
	**HDT2 vs HDT30**	**973**	**488**	**485**
	**HDT2 vs HDT60**	**793**	**500**	**293**
	HDT30 vs HDT60	0	0	0
2-effect of reambulation?	**HDT60 vs R1**	**260**	**178**	**82**
	HDT60 vs R2	50	38	12
	R1 vs R2	8	3	5
	**R1 vs R12**	**958**	**197**	**761**
	**R1 vs R30**	**467**	**158**	**309**
	R12 vs R30	1	0	1
3-recovery to baseline?	**BDC vs R30**	**326**	**275**	**51**

1-selective time point comparison using the Wald's test significance ($\alpha < 0.05$).
2-LFC > 0.05
3-LFC < 0.05
Highlighted in bold are differentially expressed genes profiles chosen for further analysis

b

Biological question	Comparison[1]	Differentially expressed protein-coding genes		
		Total	Up-regulated[2]	Down-regulated[3]
1-effect of spaceflight?	PF vs IF2	82	8	74
	IF2 vs IF3	67	64	3
	IF2 vs IF4	58	57	1
2-effect of return to Earth?	IF4 vs R1	100	95	5
	R1 vs R3	2	2	0
	R1 vs R4	100	8	92
3-return to pre-flight?	PF vs R4	0	0	0

1-selective time point comparison using the Wald's test significance ($\alpha < 0.05$).
2-LFC > 0.05
3-LFC < 0.05

Supplementary Table 2 | Summary of identified differentially expressed genes at selected time points.

We interrogated the leukocyte transcriptome of bed rest participants and astronauts with the same 3 biological questions: 1) What is the effect of prolonged exposure to bedrest or to space?; 2) What is the effect of reambulation after prolonged bed rest HDT (60 days) or landing from space (6 months)?; How complete is the recovery from prolonged bed rest HDT and spaceflight? This Table shows the differentially expressed genes at selected time points for (a) the 60-day bed rest HDT study and (b) astronaut study addressing the 3 questions.

Supplementary Figure 1

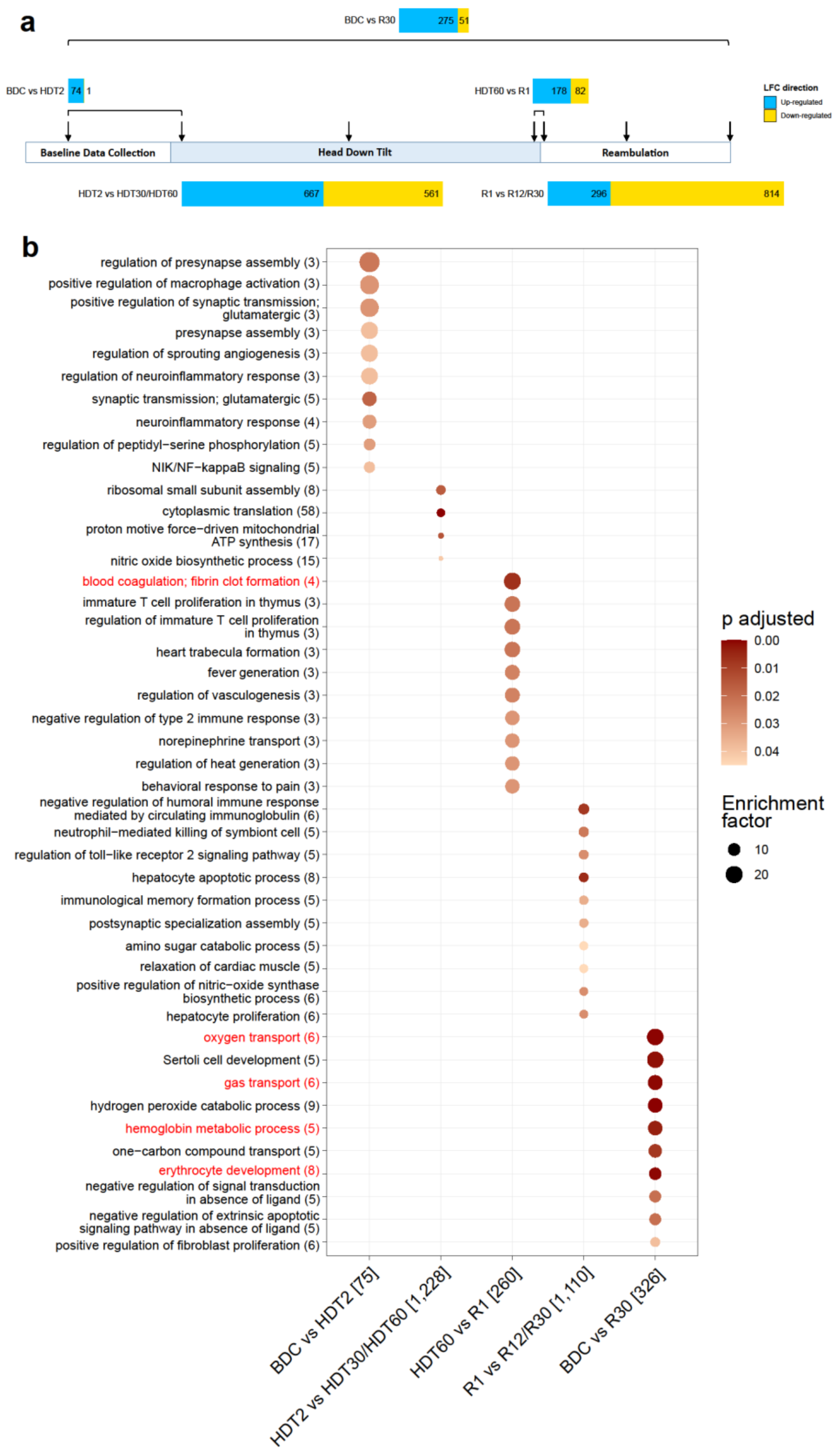

Supplementary Figure 1 | Enrichment analysis of transcriptomic changes at selected timepoint comparisons during bed rest. (a) Number of protein-coding genes differentially expressed at selected timepoint comparisons. The 3 phases of the study indicated in the study timeline: BDC (baseline data collection); HDT (head down tilt -6°); R (reambulation). Arrows indicate blood samples and collection days. Square brackets indicate the time points, BDC, HDT(Day), and R(Day), used in transcriptome comparisons to identify differentially expressed genes. (b) Dot plot displaying Gene Ontology (GO) terms obtained from the unsigned over-representation analysis (ORA) of the 5 lists of differentially expressed protein-coding gene profiles from Figure 1. Square brackets [] indicate the number of differentially expressed genes in each list. Round brackets () indicate the number of genes mapping to a specific GO term. The size of each dot is proportional to the enrichment factor (size scale) and dot colors represents the false discovery rate (FDR) adjusted p-values <0.05, where darker colors are lower values (color scale). Enrichment corresponds to the ratio of mapped gene counts to a given GO term and the reference list of 21,419 expressed genes. Erythropoiesis-related related GO terms highlighted in red.

Supplementary Figure 2

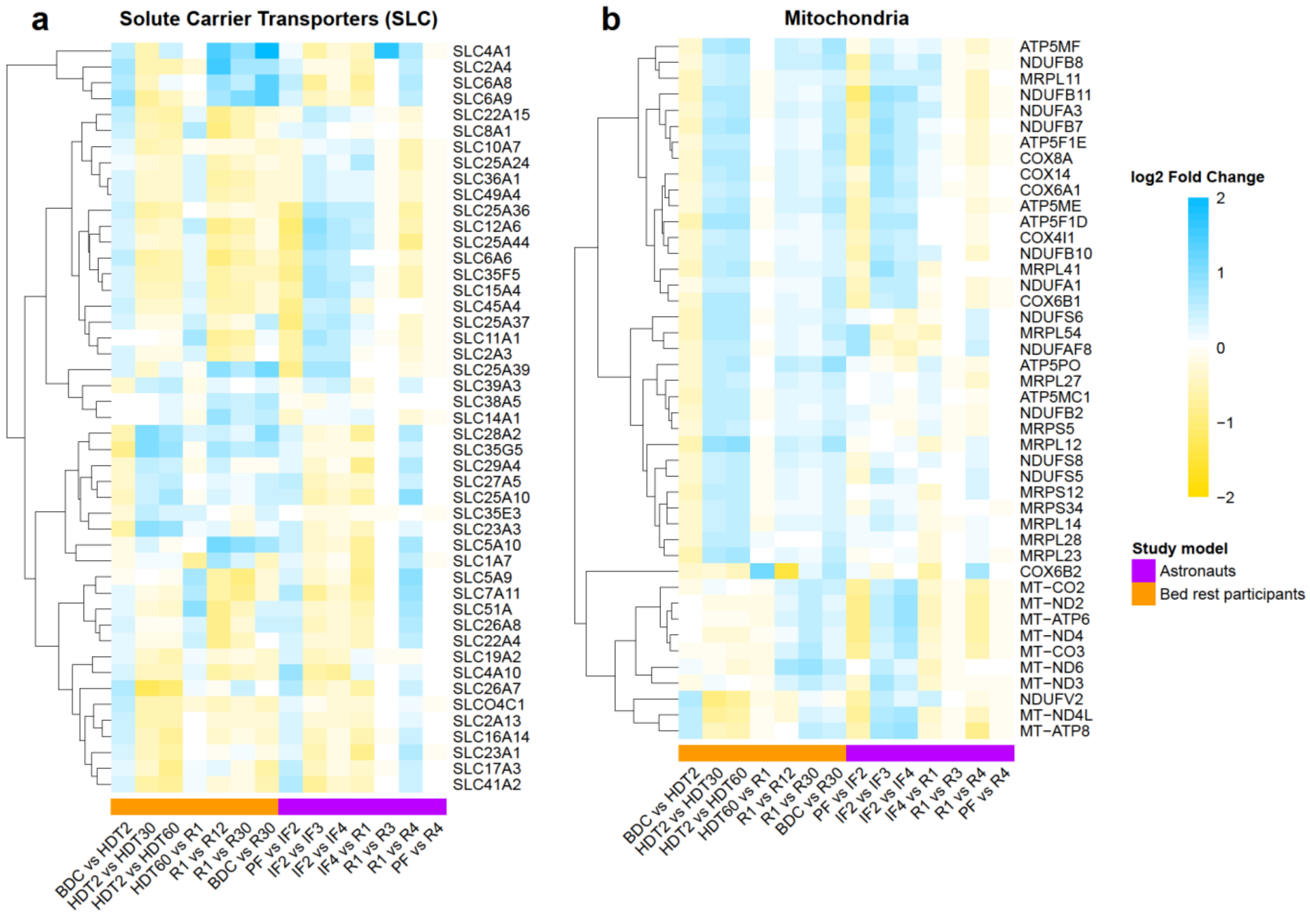

Supplementary Figure 2 | Solute carrier transporters and mitochondrial protein families represented in differentially expressed genes. Heatmaps display the log2 fold change (LFC) values for genes from the solute carrier transporters (a) and mitochondrial proteins (b). Cell colors represent the LFC values for the timepoint comparison, where blue indicates up-regulation and yellow indicates down-regulation (color scale). Bed rest data represented in the columns above the orange bar and astronaut data above the purple bar. HUGO Nomenclature Committee (HGNC) symbols were used to name genes. Dendrograms grouped genes with similar temporal profiles of changes. The figures display coordinated gene expression changes in solute carrier transporters and mitochondrial proteins at phase transitions to and from bed rest and to and from space.
